# Supplementary material for: Joint stereo 3D object detection and implicit surface reconstruction
Source: Sci Rep. 2024 Jun 17;14:13893. doi: 10.1038/s41598-024-64677-2 (PMC11183249; doi:10.1038/s41598-024-64677-2)
Supplement: Supplementary file 1 — Supplementary Information. [file 41598_2024_64677_MOESM1_ESM.pdf]

This is the supplementary material for *Joint Stereo 3D Object Detection and Implicit Surface Reconstruction*, which contains more details about the model hyper-parameters and the training setting.

1 Network architecture

The detection network used in the experiments is visualized in Fig. 3 of the main text. It has a similar architecture as LIGA[1]. Its detailed hyper-parameters are shown below.

Layer/sub-module Name	Type	Input Name:Size	Output Name:Size
Backbone	BasicBlock×[3,4,6,3]	$\mathcal{L}/\mathcal{R}$:1248×384×3	$\mathcal{L}/\mathcal{R}^F$:312×96×32
Construct Cost Volume	Interpolation	$\mathcal{L}/\mathcal{R}^F$:312×96×32	$\mathcal{C}^g$:312×96×72×64
CV-1	Conv3D-BN-ReLU, k=3, p=1, s=1	$\mathcal{C}^g$:312×96×72×64	CV^1:312×96×72×64
CV-2	Conv3D-BN-ReLU-R, k=3, p=1, s=1	CV^1:312×96×72×64	CV^2:312×96×72×64
CV-Hg-1	Conv3D-BN-ReLU, k=3, p=1, s=2	CV^2:312×96×72×64	hg^1:152×48×36×128
CV-Hg-2	Conv3D-BN-ReLU, k=3, p=1, s=1	hg^1:152×48×36×64	hg^2:152×48×36×128
CV-Hg-3	Conv3D-BN-ReLU, k=3, p=1, s=2	hg^2:152×48×36×64	hg^3:76×24×18×128
CV-Hg-4	Conv3D-BN-ReLU, k=3, p=1, s=1	hg^3:76×24×18×128	hg^4:76×24×18×128
CV-Hg-5	ConvTrans3D-BN-ReLU, k=3, p=1, s=2	hg^4,hg^2:76×24×12×128	hg^5:152×48×36×128
CV-Hg-6	ConvTrans3D-BN-ReLU, k=3, p=1, s=2	hg^5:152×48×36×128	$\mathcal{C}^{gf}$:312×96×72×64
CV-cls-1	Conv3D-BN-ReLU, k=3, p=1, s=1	$\mathcal{C}^{gf}$:312×96×72×64	cls^1:312×96×72×64
CV-cls-2	Conv3D, k=3, p=1, s=1	cls^1:312×96×72×64	cls^2:312×96×72×1
Depth Estimation	Upsample×4, Softmax	cls^2:312×96×72×1	$depth$:1248×384×1
Voxel sampling	Bilinear interpolation/Concatenation	hg^6:312×96×72×64	G:288×20×304×64
G-1	Conv3D-BN-ReLU, k=3, p=1, s=1	G:288×20×304×64	G^1:288×20×304×64
G-Hg-1	Conv3D-BN-ReLU, k=3, p=1, s=2	G^1:288×20×304×64	G^1_{hg}:144×10×152×128
G-Hg-2	Conv3D-BN-ReLU, k=3, p=1, s=1	G^1_{hg}:144×10×152×128	G^2_{hg}:144×10×152×128
G-Hg-3	Conv3D-BN-ReLU, k=3, p=1, s=2	G^2_{hg}:144×10×152×128	G^3_{hg}:72×5×76×128
G-Hg-4	Conv3D-BN-ReLU, k=3, p=1, s=1	G^3_{hg}:72×5×76×128	G^4_{hg}:72×5×76×128
G-Hg-5	ConvTrans3D-BN-ReLU, k=3, p=1, s=2	G^4_{hg},G^2_{hg}:72×5×76×128	G^5_{hg}:144×10×152×128
G-Hg-6	ConvTrans3D-BN-ReLU, k=3, p=1, s=2	G^5_{hg}:144×10×152×128	G^6_{hg}:288×20×304×64
G-pool	3D Average Pooling, k=(4,1,1), s=(4,1,1)	G^6_{hg}:288×20×304×64	G^{pool}:288×5×304×64
G-reshape	Tensor Reshape,	G^{pool}:288×5×304×64	G_{BEV}:288×304×320
BEV-1	Conv2D-BN-ReLU,	G_{BEV}:288×304×320	G^1_{BEV}:288×304×128
G-Hg-BEV-1	Conv2D-BN-ReLU, k=3, p=1, s=2	G^1_{BEV}:288×304×128	hg^1_{BEV}:144×152×256
G-Hg-BEV-2	Conv2D-BN-ReLU, k=3, p=1, s=1	hg^1_{BEV}:144×152×256	hg^2_{BEV}:144×152×256
G-Hg-BEV-3	Conv2D-BN-ReLU, k=3, p=1, s=2	hg^2_{BEV}:144×152×256	hg^3_{BEV}:72×76×256
G-Hg-BEV-4	Conv2D-BN-ReLU, k=3, p=1, s=1	hg^3_{BEV}:72×76×256	hg^4_{BEV}:72×76×256
G-Hg-BEV-5	ConvTrans2D-BN-ReLU, k=3, p=1, s=2	hg^4_{BEV},hg^2_{BEV}:72×76×256	hg^5_{BEV}:144×152×256
G-Hg-BEV-6	ConvTrans2D-BN-ReLU, k=3, p=1, s=2	hg^5:144×152×256	hg^6_{BEV}:288×304×128
BEV-cls-1	Conv2D-BN-ReLU, k=3, p=1, s=1	hg^6_{BEV}:288×304×128	G^{cls1}_{BEV}:288×304×128
BEV-cls-2	Conv2D-BN-ReLU, k=3, p=1, s=1	G^{cls1}_{BEV}:288×304×128	G^{cls2}_{BEV}:288×304×128
BEV-reg-1	Conv2D-BN-ReLU, k=3, p=1, s=1	hg^6_{BEV}:288×304×128	G^{reg1}_{BEV}:288×304×128
BEV-reg-2	Conv2D-BN-ReLU, k=3, p=1, s=1	G^{reg1}_{BEV}:288×304×128	G^{reg2}_{BEV}:288×304×128
Anchor Classification	Conv2D-BN-ReLU, k=3, p=1, s=1	G^{cls2}_{BEV}:288×304×128	cls:288×304×12
Anchor Regression	Conv2D-BN-ReLU, k=3, p=1, s=1	G^{reg2}_{BEV}:288×304×128	reg:288×304×30

Figure S1. Detailed architecture of the proposal model. Conv3D-BN- ReLU is a 3D convolution layer followed by batch normalization and ReLU activation. ConvTrans3D denotes a transposed 3D convolution layer. Conv3D-BN-ReLU-R means the output is added to the input as the residual. k denotes kernel size. p denotes padding. s denotes stride. d denotes dilation. Hg denotes the hourglass sub-module. BasicBlock denotes a basic block in ResNet.

The shape estimation branch in Ego-Net++ is visualized in Fig. 3 in the main text. It has learnable parameters $\{Ha, V, E\}$. The detailed hyper- parameters of V shown in Figure S2., which consists of 2D feature extraction, cost volume construction and processing, and depth/mask prediction. The detailed hyper-parameters of Ha, E, and the shape decoder are shown in Figure S3, Figure S4, and Figure S5 respectively.

2 Training details

The supervision for Ego-Net++ consists of the orientation estimation part and the implicit shape estimation part. The training loss of the orientation estimation part is the same as Ego-Net[5] and interested readers can refer to the official repository for more details. For the implicit shape estimation branch, the training loss consists of a cross-entropy segmentation loss L_{seg}, a smooth L_1 disparity estimation loss L_{disp} following[6], and a hallucination loss L_{Ha} as

$$L_{shape} = L_{seg} + L_{disp} + L_{Ha}. \tag{1}$$

Layer/sub-module Name	Type	Input Name:Size	Output Name:Size
Backbone-1	Conv2D-BN-ReLU, k=3, p=1, s=2	$\mathcal{L}/\mathcal{R}-RoI$:224×224×3	$F1$:112×112×32
Backbone-2	Conv2D-BN-ReLU, k=3, p=1, s=1	$F1$:112×112×32	$F2$:112×112×32
Backbone-3	Conv2D-BN-ReLU, k=3, p=1, s=1	$F2$:112×112×32	$F3$:112×112×32
Backbone-4	BasicBlock × 3, k=3, p=1, s=1	$F3$:112×112×32	$F4$:112×112×32
Backbone-5	BasicBlock × 16, k=3, p=1, s=1	$F4$:112×112×32	$F5$:56×56×64
Backbone-6	BasicBlock × 3, k=3, p=1, s=1	$F5$:56×56×32	$F6$:56×56×128
Backbone-7	BasicBlock × 3, k=3, p=1, s=1	$F6$:56×56×32	$F7$:56×56×128
Fusion	SPP-Module	$F4$-$F7$	$\mathcal{L}/\mathcal{R}^F$:56×56×32
Cost Volume Construction	Concatenation	$\mathcal{L}/\mathcal{R}^F$	$\mathcal{C}^l$:56×56×48×64
CV-1	Conv3D-BN-ReLU, k=3, p=1, s=1	$\mathcal{C}^l$:56×56×48×64	CV^1:56×56×48×32
CV-2	Conv3D-BN-ReLU-R, k=3, p=1, s=1	CV^1:56×56×48×32	CV^2:56×56×48×32
CV-3	Conv3D-BN-ReLU-R, k=3, p=1, s=1	CV^2:56×56×48×32	CV^3:56×56×48×32
CV-Hg-1	Conv3D-BN-ReLU, k=3, p=1, s=2	CV^2:56×56×48×32	hg^1:28×28×24×64
CV-Hg-2	Conv3D-BN-ReLU, k=3, p=1, s=1	hg^1:28×28×24×64	hg^2:28×28×24×64
CV-Hg-3	Conv3D-BN-ReLU, k=3, p=1, s=2	hg^2:28×28×24×64	hg^3:14×14×12×64
CV-Hg-4	Conv3D-BN-ReLU, k=3, p=1, s=1	hg^3:14×14×12×64	hg^4:14×14×12×64
CV-Hg-5	ConvTrans3D-BN-ReLU, k=3, p=1, s=2	hg^4,hg^2:14×14×12×64	hg^5:28×28×24×64
CV-Hg-6	ConvTrans3D-BN-ReLU, k=3, p=1, s=2	hg^5:28×28×24×64	hg^6:56×56×48×32
Mask-1	Conv2D-BN-ReLU, k=3, p=1, s=1	$F7$:56×56×128	M-1:56×56×128
Mask-2	Conv2D-BN-ReLU, k=3, p=1, s=1	M-1:56×56×128	M-2:56×56×128
Mask-3	ConvTrans2D-BN-ReLU, k=3, p=1, s=2	M-2:56×56×128	M-3:112×112×128
Mask prediction	ConvTrans2D-BN-ReLU, k=3, p=1, s=2	M-3:112×112×128	$\mathcal{M}$:224×224×1
Disparity-1	Upsample	hg^6:56×56×48×32	D-1:224×224×192×32
Disparity-2	Conv3D-BN-ReLU, k=1, p=0, s=1	D-1:224×224×192×32	D-2:224×224×192×1
Disparity prediction	Soft-argmax	D-2:224×224×192×1	$\mathcal{D}$:224×224×1

Figure S2. Detailed architecture of the visible surface extraction module V. Conv3D-BN-ReLU is a 3D convolution layer followed by batch normalization and ReLU activation. ConvTrans3D denotes a transposed 3D convolution layer. Conv3D-BN-ReLU-R means the output is added to the input as the residual. k denotes kernel size. p denotes padding. s denotes stride. d denotes dilation. SPP-Module refers to the pyramid module in[2].

Layer/sub-module Name	Type	Input Name:Size	Output Name:Size
PointFeat-1	Linear layer	$O(V(b_q))$:2048×3	2048×8
PointFeat-2	DGC layer	2048×8	2048×8
PointFeat-3	DGC layer	2048×8	2048×32
PointFeat-4	DGC layer	2048×32	512×64
PointFeat-5	DGC layer	512×64	512×64
PointFeat-6	DGC layer	512×64	128×128
Sequence transformation	Transformer module	2048×8	224×384
Output	Folding Net	224×384	16384×3

Figure S3. Detailed architecture of the unseen surface hallucination module Ha. The DGC layer denotes a dynamical graphical convolution layer. The transformer module and the folding net modules follows[3] and[4] respectively.

Layer/sub-module Name	Type	Input Name:Size	Output Name:Size
PointFeat-1	Linear layer	$H(O(V(b_q)))$:16384×3	F1:16384×1024
PointFeat-2	BasicBlock × 4	16384×1024	F2:16384×512
Pool-1	Maxpool × 4	F2:16384×512	P1:1×512
Repeat	Copy	P1:1×512	R:16384×512
Cat	Concatenate	R+F2	C:16384×1024
PointFeat-3	BasicBlock × 1	C:16384×1024	F3:16384×512
Pool-2	Max Pool	F3:16384×1024	P2:1×512
Prediction	Linear	P2×512	s_q:1×512

Figure S4. Detailed architecture of the point encoder module.

In implementation, we train V and Ha separately. V is trained with $L_{seg} + L_{disp}$. We train with a batch size of 16 instances for 50 epochs. Adam optimizer is used and the learning rate is 0.001. For training Ha we use ShapeNet training set as[3]. The hallucination loss is a Chamfer Distance loss between the predicted and ground truth point clouds. The training adopts a batch

Layer/sub-module Name	Type	Input Name:Size	Output Name:Size
GridFeat-1	Linear layer	$E(H(O(V(b_q))))$:16384×3 + Grid:G×3	GF1:G×512
GridFeat-2	BasicBlock-CBN × 5	GF1:G×512	GF2:16384×512
Occupancy Prediction	Linear	GF2:G×512	g_j:G×1

Figure S5. Detailed architecture of the shape decoder module. BasicBlock-CBN is a Resnet basic block with conditional batch normalization.

size of 50 and lasts 300 epochs. The learning rate starts at 0.001 and is multiplied by 0.9 after every 50 epochs. The experiments are conducted on NVIDIA RTX 3090 GPUs.

The training process of the proposal model follows LIGA[1].

References

1. Guo, X., Shi, S., Wang, X. & Li, H. Liga-stereo: Learning lidar geometry aware representations for stereo-based 3d detector. In *Proceedings of the IEEE/CVF International Conference on Computer Vision*, 3153–3163 (2021).
2. Chang, J.-R. & Chen, Y.-S. Pyramid stereo matching network. In *Proceedings of the IEEE Conference on Computer Vision and Pattern Recognition*, 5410–5418 (2018).
3. Yu, X. *et al.* Pointr: Diverse point cloud completion with geometry-aware transformers. In *Proceedings of the IEEE/CVF International Conference on Computer Vision*, 12498–12507 (2021).
4. Yang, Y., Feng, C., Shen, Y. & Tian, D. Foldingnet: Interpretable unsupervised learning on 3d point clouds. *arXiv preprint arXiv:1712.07262* **2**, 5 (2017).
5. Li, S., Yan, Z., Li, H. & Cheng, K.-T. Exploring intermediate representation for monocular vehicle pose estimation. In *Proceedings of the IEEE/CVF Conference on Computer Vision and Pattern Recognition*, 1873–1883 (2021).
6. Sun, J. *et al.* Disp r-cnn: Stereo 3d object detection via shape prior guided instance disparity estimation. In *Proceedings of the IEEE/CVF Conference on Computer Vision and Pattern Recognition*, 10548–10557 (2020).
